# Thyroid Nodule with Benign Cytology: Is Clinical Follow-Up Enough?

**DOI:** 10.1371/journal.pone.0063834

**Published:** 2013-05-24

**Authors:** Yoon Jung Choi, Inkyung Jung, Sung Ji Min, Hye Jung Kim, Ji-hoon Kim, Soojin Kim, Jeong Seon Park, Jung Hee Shin, Yu-Mee Sohn, Jung Hyun Yoon, Jin Young Kwak

**Affiliations:** 1 Department of Radiology, Kangbuk Samsung Hospital, Sungkyunkwan University, Seoul, South Korea; 2 Department of Biostatistics, Yonsei University College of Medicine, Seoul, South Korea; 3 Graduate School of Health and Welfare CHA University, Seongnam, South Korea; 4 Department of Radiology, Kyungpook National University Medical Center, Seoul, South Korea; 5 Department of Radiology, Seoul National University Hospital, Seoul, South Korea; 6 Department of Radiology, Chung-Ang University Hospital, Seoul, South Korea; 7 Department of Radiology, Hanyang University Hospital, Hanyang University College of Medicine, Seoul, South Korea; 8 Department of Radiology and Center for Imaging Science, Samsung Medical Center, Sungkyunkwan University School of Medicine, Seoul, South Korea; 9 Department of Radiology, Kyung Hee University Hospital, College of Medicine, Kyung Hee University, Seoul, South Korea; 10 Department of Radiology, CHA Bundang Medical Center, CHA University College of Medicine, Seongnam, South Korea; 11 Department of Radiology, Severance Hospital, Research Institute of Radiological Science, Yonsei University College of Medicine, Seoul, South Korea; University of Nebraska Medical Center, United States of America

## Abstract

**Objective:**

In this multicenter study, we investigated the management algorithm for thyroid nodules with benign cytology using US features from data collected from 7 institutions.

**Materials and Methods:**

The institutional review board approved this retrospective study. Data on 700 focal thyroid nodules in 673 consecutive patients were collected from 7 university-affiliated hospitals. This study included nodules that were diagnosed as benign at initial cytologic evaluation and that underwent pathologic or follow-up study. The risk of malignancy was compared according to the US assessments of each institution as well as looking at all the nodules together as a whole.

**Results:**

Of the 700 nodules, 688 (98.3%) were benign and 12 (1.7%) were malignant. If initial cytologic results were benign, the likelihood of the nodule actually being malignant was from 1 to 3%, varying by institution. The likelihood of a cytologically benign nodule with positive US being malignant (4.7%, 8/169) was higher than that of one without positive US (0.8%, 4/531) (*P* = .002).

**Conclusion:**

Based on our multicenter study, repeat FNA should be performed in thyroid nodules with initial benign cytology showing suspicious US features in order to decrease the number of false negative cases.

## Introduction

In the diagnosis of focal thyroid lesions, ultrasound (US) and US-guided fine-needle aspiration (US-FNA) have become widely used, routine procedures that decide the direction of further management [1]–[4]. Although US-FNA is an operator-dependent diagnostic tool, it definitely shows higher diagnostic accuracy compared to palpation-guided FNA [5]. Also US shows relatively good interobserver agreement in reaching a final assessment, especially among experienced physicians [6]–[8]. A thyroid nodule with benign cytology carries about 0–3% risk of malignancy, thus clinical follow up is recommended over repeat FNA [9]–[11]. However, the risk of malignancy in a thyroid nodule with benign cytology varies among institutions, with reported values being between a wide range of 2% to 18% [12]. Therefore, recommendations in the management of these nodules vary from routine follow up to repeat FNA. Although some papers have demonstrated a selection approach based on US features for repeat FNA in benign thyroid nodules [13]–[15], whether this approach may be generally reproducible in other institutions has not been verified. In this multicenter study, we retrospectively investigated the management algorithm for thyroid nodules with benign cytology using US features from data collected from 7 different institutions.

## Materials and Methods

The institutional review board of severance hospital approved of this retrospective observational study and required neither patient approval nor informed consent for our review of patients’ images and patients’medical records. However, written informed consent was obtained from all patients for US-FNA prior to each procedure as a daily practice.

### Study Population

Study subjects were collected from 7 institutions of the Korean Society of Thyroid Radiology group. Seven radiologists with more than 3 years of experience in thyroid imaging participated in patient collection and review. Each institution contributed 100 consecutive thyroid nodules equal to or larger than 1 cm with benign cytology at initial FNA for this multicenter study. Patients were included according to the following criteria: (a) patients who underwent thyroid surgery, (b) patients who had at least 2 consecutive US-FNA sessions in which both results showed benign cytology, and (c) patients with benign initial cytology who had no interval change or decreased size on follow-up US performed at least 1 year from March 2007. After applying the inclusion criteria, this study finally included 700 thyroid nodules in 673 patients. Size increase was defined as more than 50% increase in volume or more than 20% increase in at least two dimensions that showed a minimum 2 mm increase in solid nodules or the solid portion in mixed cystic–solid nodules [9], [15]–[19].

Patient age ranged from 11 to 91 years (mean, 52.3 years). Mean age was 54.3 years (range, 11–84 years) in men, and 52 years (range, 13–91 years) in women. Mean nodule size was 20.3±10.5 mm (range, 10–49 mm).

### Imaging and Imaging Analyses

To evaluate thyroid nodules, US images were taken using various US machines with high frequency linear array transducers. Scanning protocol in all cases included both transverse and longitudinal real-time imaging of thyroid nodules, with the use of representative Digital Imaging and Communications in Medicine (DICOM) images.

For analysis of US images, participating radiologists were asked to assess thyroid nodules according to criteria from previously published literature [9]–[11], [20]–[26]. The criteria used in analysis included the echogenicity of solid portion, orientation, shape, margin, and calcifications. Echogenicity was classified as hyper-, iso-, or hypoechogenicity (when a nodule showed hyper-, iso-, or hypoechogenicity compared to the normal thyroid gland), or marked hypoechogenicity (when a nodule showed relative hypoechogenicity compared to the surrounding strap muscle). Margin was classified as well defined or not-well defined (microlobulated or irregular margin). Calcifications were classified as microcalcification (equal to or less than 1 mm in diameter; tiny, punctate, hyperechoic foci, either with or without acoustic shadows), macrocalcification including rim or eggshell calcification, or no calcification. When the nodules had both types of calcifications (macrocalcifications including rim calcifications intermingled with microcalcifications), the nodule was considered to have microcalcifications. Shape was classified as wider than tall or taller than wide. Suspicious malignant gray scale US features included marked hypoechogenicity, not-well defined margin, microcalcifications, and taller than wide shape. When thyroid nodules showed one or more of the above suspicious malignant gray scale US features, they were assessed as “positive”. When thyroid nodules showed no suspicious features, they were assessed as “negative” [15], [17]–[19], [21].

### Data and Statistical Analysis

Histopathologic results from surgical pathology or repeat US-FNA with follow-up US were used as standard reference. Categorical data were summarized using frequencies and percentages. We compared patients with benign or malignant thyroid nodules according to gender and US grouping using Fisher’s exact test. The independent two-sample *t*-test was used to evaluate the association between patient age or nodule size to malignancy. Malignancy rate was compared according to US groupings using Fisher’s exact test.

Analysis was performed with SAS software (version 9.2; SAS Institute, Cary, NC). Statistical significance was assumed when the *P* value was less than 0.05. All reported *P* values are 2-sided.

## Results

Table 1 shows the baseline characteristics of the patients included in this study. Age, gender, and tumor size were not significantly different between malignant and benign nodules. However, US assessments were significantly different between malignant and benign nodules (*P = *.002).

**Table 1:** Baseline characteristics of 700 thyroid nodules in 673 patients.

	Malignant(n = 12)	Benign(n = 688)	*P*
Mean age (years)	52.7±9.7	52.3±12.2	0.907
Gender			1
Men	1 (8.3%)	85 (12.4%)	
Women	11 (91.7%)	603 (87.6%)	
Mean nodule size (mm)	20.3±18.3	20.3±10.3	0.907
US assessment			0.002
Positive	8 (66.7%)	161 (76.6%)	
Negative	4 (33.3%)	527 (23.4%)	

Of the 700 nodules included, 688 (98.3%) were benign and 12 (1.7%) were malignant. Among the total collected data, 89 cases were confirmed by surgical pathology (Table 2). Malignant rates were equal to or less than 3% in thyroid nodules with benign cytology, regardless of institution (Table 3). In nodules with benign cytology which did not have positive US, malignant rates were equal to or less than 1.5% in all 7 institutions. In 5 of the 7 institutions, malignant rates were more than 3% in nodules with positive US and were higher than those without. When analyzing all the 700 thyroid nodules with benign cytology from the 7 institutions together as a whole, the likelihood (4.7%, 8/169) of a cytologically benign nodule with positive US actually benign malignant was higher than that (0.8%, 4/531) of one without positive US (*P* = .002, Table 3).

**Table 2:** Histopathologic Results of the 89 Nodules confirmed by Surgery.

	Histopathologic result	Number	Percentage
Malignant (n = 12)	Papillary carcinoma	6	50
	Follicular variant of papillary carcinoma	3	25
	Minimally invasive follicular carcinoma	2	16.7
	Minimally invasive Hurthle cell carcinoma	1	8.3
Benign (n = 77)	Adenomatous hyperplasia	66	85.7
	Follicular adenoma	7	9.1
	Hashimoto’s thyroiditis	4	5.2

**Table 3:** Likelihood of a Thyroid Nodule with Initial Benign Cytology Being Malignant According to each Institution.

Institutions	Likelihood of NoduleBeing Malignant (%)	Likelihood of Nodule withNegative US Being Malignant (%)	Likelihood of Nodule withPositive US Being Malignant (%)	*P* value
A	2	0 (0/93)	28.6 (2/7)	0.004
B	1	1.5 (1/68)	0 (0/32)	1
C	2	0 (0/37)	3.2 (2/63)	0.529
D	1	1 (1/96)	0 (0/4)	1
E	2	1.3 (1/78)	4.5 (1/22)	0.393
F	3	1.3 (1/75)	8 (2/25)	0.153
G	1	0 (0/84)	6.3 (1/16)	0.16
Total	12	0.8 (4/531)	4.7 (8/169)	0.002

## Discussion

There are several management approaches accepted for thyroid nodules with benign cytology [27]–[30]. The American Thyroid Association (ATA) recommends that all benign thyroid nodules be followed with serial US examinations performed 6–18 months after the initial FNA [9]. If the nodule size is stable (i.e., no more than a 50% change in volume or less than 20% increase in at least two nodule dimensions in solid nodules or in the solid portion of mixed cystic–solid nodules), the interval before the next clinical examination or US may be longer, e.g., every 3–5 years. If there is evidence for nodule growth either by palpation or US (more than a 50% change in volume or a 20% increase in at least two nodule dimensions with a minimal increase of 2 mm in solid nodules or in the solid portion of mixed cystic–solid nodules), FNA should be repeated, preferably with US guidance. The American Association Clinical Endocrinologists/Associazione Medici Endocrinologi (AACE/AME) suggests only simple clinical follow-up without US in patients with thyroid nodules with benign cytology as long as they do not present with clinical problems [11]. The Bethesda guidelines also suggest that as the malignant rate of benign cytology can be less than 3% [31], clinical follow-up may be enough for further evaluation. These recommendations are based on the belief that a thyroid nodule with benign cytology has a very low malignant rate. However, false negative rates of thyroid nodules with benign cytology have been reported to be relatively higher (more than 10%) than expected in some institutions [32], [33], supporting the necessity of repeat FNA in nodules with benign cytology.

Recently, several reports have proposed combining US and cytology results in management decisions of thyroid nodules, and repeat FNA may be selectively performed in thyroid nodules with benign cytology which show suspicious US features [13]–[15], [19], [28]. Investigations into selecting repeat FNA as a management approach for thyroid nodules with benign cytology using US features have been done and reported on in a few institutions [13], [28], [34], [35]. Therefore, we investigated the management algorithm for thyroid nodules with benign cytology using US features from data collected from multiple institutions. This study demonstrated a malignancy rate of 1.7% in thyroid nodules with initial benign cytology, and most institutions showed higher malignancy rates in nodules showing suspicious US features (0–28.6%) compared to those without any suspicious US features (0–1.5%). With the provided data of our multicenter study, it makes sense to select nodules according to US features, resulting in reduction of unnecessary repeat FNAs, while not overlooking malignancy among thyroid nodules with benign cytology on initial FNAs.

There were several limitations to this study. First, selection bias may have existed when choosing patients to enroll in this study. Patients who had not undergone follow-up after initial benign cytology or surgery after malignant cytology results were not included in this study. Second, the interobserver variability in US image interpretation among the 7 radiologists was not evaluated. In a multicenter study, several factors such as using different US equipment, as well as an inevitable interobserver variability can affect the final assessment of thyroid nodules. In order to minimize this limitation, the same reference standards published in previous reports were used [17], [20], [23]. Third, when we compared malignant rates according to US positive and US negative among the 7 institutions, only 1 institution had a significantly higher malignancy rate, while others did not show significant differences. This may be due to the small skewed sample size. The total number of higher malignant rates was statistically significant in nodules with positive US.

In conclusion, based on our multicenter study, repeat FNA should be performed in thyroid nodules with initial benign cytology showing suspicious US features in order to decrease the number of false negative cases.
